# Effects of nandrolone decanoate on lean body mass and anemia in adults with chronic kidney disease: a systematic review and meta-analysis

**DOI:** 10.1007/s12020-026-04771-9

**Published:** 2026-09-09

**Authors:** Lucas Caseri Câmara, Diogo Pinto da Costa Viana, Lúcio de Sousa Monte Alto, Samara Oliveira Mattoso, Nelson Carvas Junior, Matheus Hissa Lourenço Ferreira, Zahid Khan

**Affiliations:** 1 Department of Clinical Anabolism, College of Governance, Engineering and Education of São Paulo (FGE-SP), São Paulo, Brazil; 2 Brazilian Society of Endocrinology and Metabolism in Sports and Exercise, São Paulo, Brazil; 3 Newton Paiva University Center, Belo Horizonte, MG Brazil; 4 Department of Postgraduate Studies in Evidence-Based Health, Federal University of São Paulo (UNIFESP), São Paulo, Brazil; 5 Federal University of Minas Gerais (UFMG), Belo Horizonte, Brazil; 6 Townsville University Hospital, Townsville, QLD Australia; 7 James Cook University, Townsville, QLD Australia; 8 Queen Mary University of London, London, UK

**Keywords:** Chronic kidney disease, Nandrolone decanoate, Sarcopenia, Anemia, Lean body mass

## Abstract

**Purpose:**

Protein-energy wasting, sarcopenia, and anemia are common complications of chronic kidney disease (CKD) and are associated with poor outcomes. This systematic review and meta-analysis evaluated the effects of nandrolone decanoate on lean body mass and hematologic parameters in adults with CKD.

**Methods:**

We searched PubMed/MEDLINE, CENTRAL, Embase, and LILACS up to February 18, 2026, for randomized controlled trials of intramuscular nandrolone decanoate in adults with CKD. Primary outcomes were lean body mass, hemoglobin, and hematocrit. Risk of bias and certainty of evidence were assessed using RoB 2 and GRADE, respectively.

**Results:**

Fifteen RCTs published between 1974 and 2021 were included. Nandrolone decanoate was associated with increased lean body mass (4 trials; n = 125; MD 2.63 kg, 95% CI 1.60–3.65). Pooled estimates for hemoglobin (6 trials; n = 239; MD 0.81 g/dL) and hematocrit (7 trials; n = 198; MD 3.12%) also favored nandrolone, but were limited by substantial heterogeneity and very low certainty of evidence, precluding firm conclusions. Quality-of-life effects were inconsistent, and long-term safety data were sparse.

**Conclusion:**

Nandrolone decanoate was associated with increased lean body mass in adults with CKD. However, the evidence base is largely historical, and substantial heterogeneity and very low certainty of evidence preclude firm conclusions regarding hematologic benefits. Contemporary randomized trials are needed to clarify whether nandrolone has any role in current CKD management.

**Supplementary Information:**

The online version contains supplementary material available at https://doi.org/10.1007/s12020-026-04771-9.

## Introduction

Chronic kidney disease (CKD) affects approximately 9–13% of the global population and remains a major contributor to morbidity and premature mortality worldwide [1]. As kidney function declines, patients frequently develop protein-energy wasting and sarcopenia, characterized by progressive loss of lean body mass and impaired physical performance, both strongly associated with adverse clinical outcomes [2, 3]. Anemia is another prevalent complication in advanced stages, further exacerbating fatigue, reduced exercise capacity, and functional decline [4, 5].

Despite nutritional strategies, erythropoiesis-stimulating agents, and resistance exercise training, many individuals remain in a persistent catabolic state driven by functional hypogonadism, chronic inflammation, and anabolic resistance, which together sustain progressive muscle wasting [3, 6, 7].

In this context, nandrolone decanoate, a synthetic anabolic steroid with moderate androgenic activity, has been explored as an adjunctive therapeutic strategy aimed at stimulating muscle protein synthesis and enhancing erythropoiesis. Although its clinical application predates the widespread adoption of recombinant erythropoietin, more recent trials have revisited its potential to mitigate sarcopenia and improve body composition, particularly when combined with resistance exercise [4, 8–10].

A previous meta-analysis compared androgen therapy with erythropoietin for the treatment of anemia in chronic kidney disease; however, it focused primarily on hemoglobin outcomes, included a limited number of small trials, and did not evaluate effects on lean body mass or broader anabolic endpoints [11].

Earlier trials (1970–1980s) primarily investigated hematologic responses, reflecting limited anemia treatment options before the widespread availability of recombinant erythropoietin [12, 13]. From the late 1990s onward, studies increasingly incorporated assessments of body composition and muscle mass, paralleling recognition of protein-energy wasting and sarcopenia as key determinants of morbidity and mortality in CKD [14, 15].

This shift toward an integrated anabolic and functional perspective supports the need for a contemporary synthesis addressing both dimensions. Given the central role of muscle wasting in CKD pathophysiology [14, 16], and its association with adverse outcomes, including mortality [15], an updated meta-analysis of the dual anabolic and hematologic effects of nandrolone decanoate is warranted.

Therefore, this systematic review and meta-analysis aims to quantitatively synthesize randomized controlled trials assessing the effects of nandrolone decanoate on lean body mass and anemia-related outcomes in adults with chronic kidney disease. To our knowledge, this is the first meta-analysis specifically synthesizing the dual anabolic and hematologic effects of nandrolone decanoate exclusively in CKD populations.

## Methods

This systematic review and meta-analysis were conducted and reported in accordance with the Preferred Reporting Items for Systematic Reviews and Meta-Analyses (PRISMA) 2020 statement [17]. The completed PRISMA checklist is provided as supplementary material. A systematic search of PubMed/MEDLINE, Cochrane Library (CENTRAL), Embase, and LILACS was conducted independently by two reviewers on February 18, 2026.

The protocol for this review was prospectively registered in the International Prospective Register of Systematic Reviews (PROSPERO) under registration number CRD420261321835.

### Eligibility criteria

Studies were eligible if they were randomized controlled trials (RCTs) enrolling adults (≥18 years) with chronic kidney disease at any stage, including patients receiving maintenance hemodialysis, peritoneal dialysis, or pre-dialysis care. There were no restrictions regarding sex, baseline anemia status, disease duration, or nutritional status.

Studies were excluded if they involved pediatric populations, acute kidney injury without established chronic kidney disease, kidney transplant recipients without extractable CKD-specific data, or if they evaluated anabolic agents other than nandrolone decanoate without a separate nandrolone arm. Non-randomized studies, observational designs, case reports, reviews, conference abstracts without sufficient data, and animal or in vitro studies were also excluded.

Eligible interventions included intramuscular nandrolone decanoate administered at any dose, frequency, or duration, either as monotherapy or combined with standard care (e.g., erythropoietin therapy or resistance exercise training). Comparator groups could include placebo, standard care, erythropoietin alone, resistance exercise alone, or other active controls.

Studies were required to report at least one primary outcome (lean body mass, hemoglobin, or hematocrit). Safety outcomes and quality-of-life measures were included when available and were synthesized narratively when meta-analysis was not feasible.

For crossover trials, only data from the first treatment period were extracted when available to avoid potential carryover effects. Paired analyses were conducted only when sufficient within-participant data were reported, in accordance with methodological recommendations for the inclusion of crossover trials in meta-analyses. The eligibility criteria are summarized according to the PICO (Population, Intervention, Comparator, and Outcomes) framework in Table 1.

**Table 1:** PICO framework of the systematic review and meta-analysis

Element	Description
**Population**	Adults (≥ 18 years) with chronic kidney disease at any stage, including patients on maintenance hemodialysis, peritoneal dialysis, or pre-dialysis care. No restrictions on sex, baseline anemia status, disease duration, or nutritional status.
**Intervention**	Intramuscular nandrolone decanoate at any dose, frequency, or duration, administered either as monotherapy or in combination with standard care, such as erythropoietin therapy or resistance exercise training.
**Comparator**	Placebo, standard care alone, erythropoietin alone, resistance exercise training alone, or other active controls.
**Outcomes**	**Primary outcomes**: Lean body mass measured by DXA, hemoglobin, and hematocrit. **Secondary outcomes**: Quality of life and safety, including adverse events.

Abbreviations: *PICO* Population, Intervention, Comparator, Outcomes, *DXA*  dual-energy X-ray absorptiometry

### Information sources

A comprehensive literature search was conducted in the following electronic databases: PubMed/MEDLINE, Cochrane Central Register of Controlled Trials (CENTRAL), Embase, and LILACS. All databases were searched from inception to February 18, 2026, without restrictions on language or publication date. In addition, the reference lists of all included studies and relevant reviews were manually screened to identify additional eligible trials. Clinical trial registries, including ClinicalTrials.gov and the World Health Organization International Clinical Trials Registry Platform (WHO ICTRP), were also consulted to identify ongoing or unpublished studies.

### Search strategy

The search strategy combined controlled vocabulary (MeSH and Emtree terms) and free-text keywords related to chronic kidney disease and nandrolone decanoate, including terms for dialysis modalities and anabolic or androgenic therapy, with filters for randomized controlled trials. Strategies were adapted to each database’s indexing system. Animal-only studies were excluded using validated human filters when applicable. Full electronic search strategies are provided in Supplementary Table 1.

Study Selection.

All records identified through the database searches were imported into Rayyan [18], an online systematic review management software, for screening and duplicate removal. Two reviewers independently screened titles and abstracts against the predefined eligibility criteria. Full-text articles were subsequently retrieved and assessed independently by the same reviewers to determine final eligibility. Discrepancies at any stage of the selection process were resolved through discussion, and when necessary, consultation with a third reviewer. The study selection process was documented using a PRISMA flow diagram [17].

### Data extraction

Data extraction was performed independently by two reviewers using a standardized and piloted data collection form. Extracted information included study characteristics (author, year, country, study design, sample size), participant characteristics (age, sex, CKD stage, dialysis modality), intervention details (nandrolone dose, frequency, duration, and co-interventions), comparator characteristics, and reported outcomes.

For continuous outcomes (lean body mass, hemoglobin, and hematocrit), means, standard deviations, and sample sizes corresponding to the reported effect estimates were extracted. Change-from-baseline values were preferentially collected when available; otherwise, post-intervention values were used. The sample size entered into the meta-analysis was selected according to the analysis population underlying each extracted estimate. When an intention-to-treat (ITT) analysis was explicitly reported and corresponding summary statistics were available, the ITT sample size was preferentially used. When no formal ITT analysis was reported, we used the number of participants who contributed data to the relevant outcome analysis (per-protocol, completer, or available-case populations, as applicable). Randomized group sizes were used only when they also represented the analytical population or when the outcome-specific analytical sample size could not be determined more precisely from the publication. When necessary, standard deviations were calculated from standard errors, confidence intervals, p-values, or other available statistical information using established formulas. Authors were contacted when data were incomplete or unclear.

Disagreements in data extraction were resolved by discussion, with consultation from a third reviewer when required.

### Risk of bias assessment

The risk of bias of the included studies was independently assessed by two reviewers using the Cochrane Risk of Bias 2 (RoB 2) tool [19]. The following domains were evaluated: bias arising from the randomization process; bias due to deviations from intended interventions; bias due to missing outcome data; bias in measurement of the outcome; and bias in selection of the reported result.

Each domain was judged as “low risk of bias”, “some concerns”, or “high risk of bias”, according to the RoB 2 guidance. An overall risk of bias judgment was assigned to each study based on the domain-level assessments. Disagreements between reviewers were resolved through discussion, with consultation from a third reviewer when necessary.

### Statistical analysis

Quantitative synthesis was performed when at least two studies reported comparable data for a given outcome. For continuous outcomes (lean body mass, hemoglobin, and hematocrit), pooled effect sizes were calculated using Mean Differences (MD), as outcomes were reported in the same units. Meta-analyses were conducted using the metacont function. For each comparison, the group sample sizes entered into the meta-analysis corresponded to the analysis population associated with the extracted means and standard deviations, according to the hierarchy described in the Data Extraction section. Random-effects models were fitted using the Restricted Maximum Likelihood (REML) estimator for between-study variance. Random-effects models using REML were applied a priori because clinical and methodological heterogeneity was expected across studies, including differences in patient populations, nandrolone dosing regimens, treatment duration, and concomitant therapies. All methods followed the Cochrane Handbook recommendations [20].

For hemoglobin, subgroup analyses were prespecified according to comparator type: (i) nandrolone plus erythropoietin versus erythropoietin alone; (ii) nandrolone versus placebo/standard care; and (iii) nandrolone versus erythropoietin. A test for subgroup differences explored potential effect modification.

When change-from-baseline standard deviations were unavailable, they were imputed using a correlation coefficient of *r* = 0.7. This value was chosen as a plausible moderate-to-high within-participant correlation for repeated physiological outcomes (hemoglobin, hematocrit, and lean body mass) in stable CKD populations. It represents a balanced assumption that avoids both the overly conservative widening of confidence intervals that would result from assuming a very low correlation and the potential overprecision associated with assuming a very high correlation. The same coefficient was applied consistently across studies, following Cochrane Handbook recommendations for imputing missing change-score standard deviations when study-specific correlations are not reported [20]. For crossover trials, only data from the first treatment period were extracted when available to avoid potential carryover effects.

Sensitivity analyses were performed to evaluate the influence of crossover trials on the pooled estimates. Due to the high proportion of studies requiring imputation of change-score standard deviations, a sensitivity analysis excluding these studies was not considered informative and was not performed. Results of the sensitivity analysis excluding crossover trials are reported in the Results section.

Heterogeneity was quantified using I² and τ. I² values were interpreted using conventional thresholds (0–40% possibly unimportant; 30–60% moderate; 50–90% substantial; 75–100% considerable), recognizing that these are context dependent [20]. 95% prediction intervals were calculated to estimate the expected range of effects in future studies [20].

Forest plots were generated for all meta-analyses. Publication bias was assessed using funnel plots and Egger’s regression test [21] when ≥ 10 studies were available. Analyses were performed using R (version 4.5.1) [22] in Rstudio [23] with the meta package [24].

### Certainty of evidence

The certainty of evidence for each outcome was assessed using the Grading of Recommendations Assessment, Development and Evaluation (GRADE) approach [25]. The quality of evidence was rated as high, moderate, low, or very low based on the following domains: risk of bias, inconsistency, indirectness, imprecision, and publication bias. As all included studies were randomized controlled trials, the initial certainty level was considered high and was downgraded when limitations were identified across these domains. Summary of Findings tables were generated to present the overall certainty of evidence for the primary and secondary outcomes.

## Results

### Study selection

The database search identified a total of 86 records (PubMed: 53; Cochrane Library: 21; Embase: 10; LILACS: 2). After removal of 16 duplicate records, 70 unique studies remained for title and abstract screening. Of these, 50 articles were excluded based on title and abstract. The full texts of 20 studies were assessed for eligibility, and 15 RCTs met the inclusion criteria and were included in the final qualitative synthesis.

**Fig. 1:**
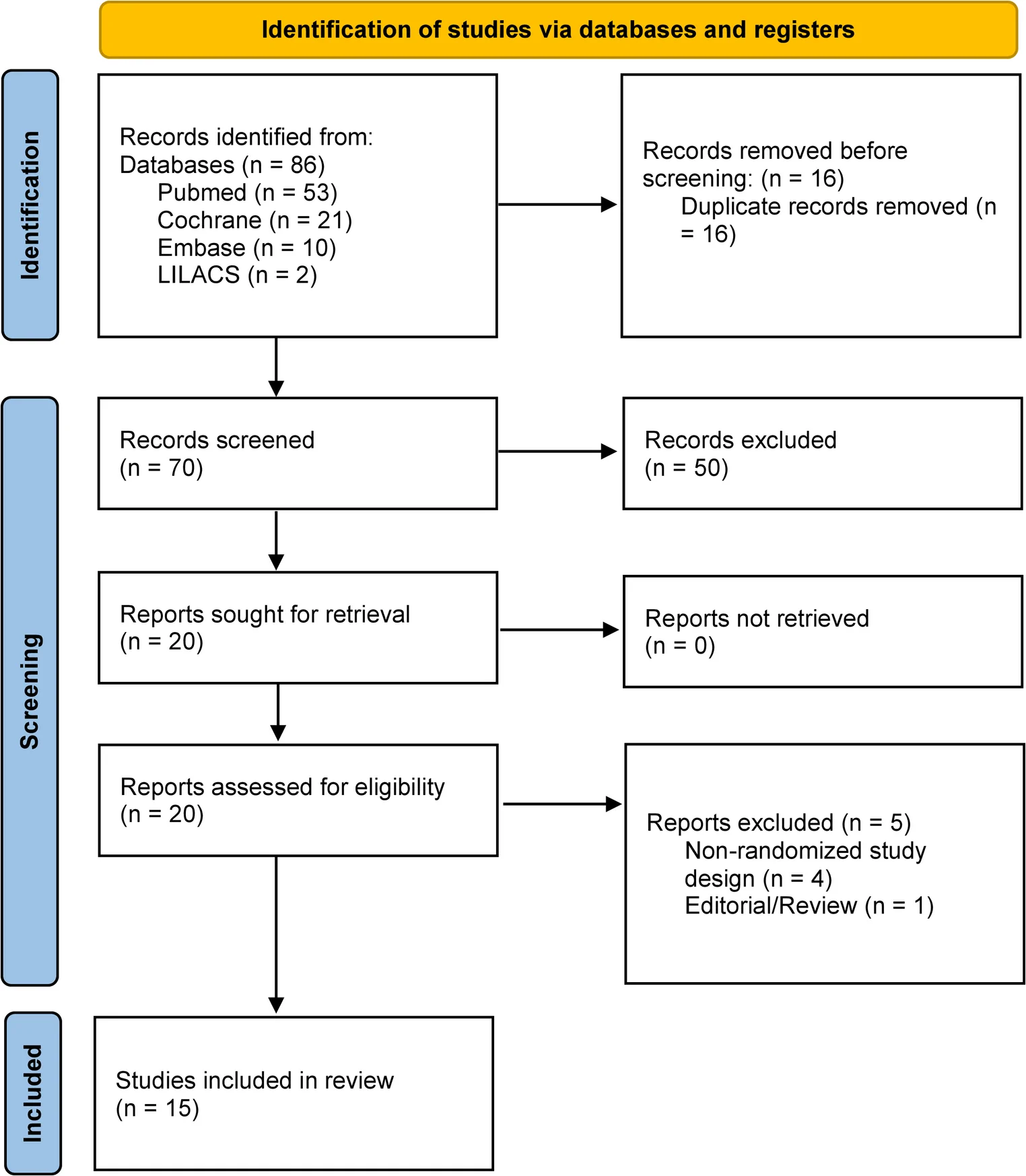
PRISMA 2020 flow diagram of the study selection process

The study selection process is presented in the PRISMA flow diagram (Fig. 1).

### Studies excluded after full-text assessment

Five studies that initially appeared to meet the inclusion criteria were excluded after full-text assessment. Doane et al. (1975) [26], Teruel et al. (1997) [27], Ballal et al. (1991) [28], and Lee et al. (2002) [29] were excluded because they were not randomized controlled trials. Johansen (2001) [30] was excluded because it was an editorial/review article and did not report original randomized data. Detailed reasons are presented in Supplementary Table S2.

### Study characteristics

The main characteristics of the 15 included randomized controlled trials are summarized in Table 2. Studies were published between 1974 and 2021 and predominantly enrolled patients on maintenance hemodialysis, with some including pre-dialysis or peritoneal dialysis populations. Nandrolone decanoate was administered intramuscularly at weekly doses ranging from 50 to 200 mg for durations of 12 weeks to 12 months (Table 2).

**Table 2:** Characteristics of randomized clinical trials evaluating nandrolone decanoate in chronic kidney disease

Author (Year)	Study Design	Population	Intervention	Comparator	Duration	Main Outcomes	Main Findings
Williams et al. 1974 [35]	Double-blind, placebo-controlled	18 chronic HD patients	ND 200 mg IM weekly	Placebo	13 weeks	Hb, Ht, red cell mass	↑ Hb (+ 1.2 g/dL), ↑ Ht (+ 4.3%), ↑ red cell mass
Buchwald et al. 1977 [31]	Double-blind crossover	21 HD patients	ND 200 mg (men) / 100 mg (women) IM weekly	Placebo	NR	Hb, Ht, EPO	↑ Hb, ↑ red cell mass
Cattran et al. 1977 [36]	Randomized crossover	37 HD men	ND 200 mg weekly	No androgen	12 months	Hb, Ht	↑ Hb/Ht in residual kidney function
Neff et al. 1981 [8]	Randomized crossover	143 HD patients	ND 3 mg/kg IM weekly	Control period (no androgen)	6–8 months periods	Ht, red cell mass	Injectable superior; ~50% response
Solomon et al. 1988 [32]	Randomized controlled trial	HD patients	ND 100 mg IM weekly	Standard care	6 months	Red cell mass	↑ RBC mass
Hendler et al. 1990 [33]	Randomized controlled trial	HD patients	ND 100 mg IM weekly	Control	6 months	RBC metabolism	↑ RBC mass; limited functional impact
Gaughan et al. 1997 [41]	Randomized controlled trial	19 HD patients	rHuEPO + ND 100 mg weekly	rHuEPO	26 weeks	Ht	Greater Ht increase in combined group
Gascón et al. 1999 [37]	Randomized controlled trial	33 elderly HD men	ND 200 mg weekly	rHuEPO	6 months	Hb, Ht	↑ Hb/Ht; improved nutrition
Johansen et al. 1999 [4]	Double-blind RCT	29 dialysis patients	ND 100 mg weekly	Placebo	6 months	Lean body mass	↑ Lean body mass
Baig et al. 2021 [38]	Randomized controlled trial	100 CKD anemia patients (50/group)	ND 100 mg IM weekly + EPO 2000 U SC 2×/week	rHuEPO	6 months	Hb rise	↑ Hb mean
Navarro et al. 2002 [39]	Randomized controlled trial	27 CAPD men	ND 200 mg weekly	rHuEPO	6 months	Hb, Ht	Similar hematologic response
Sheashaa et al. 2005 [40]	Randomized controlled trial	32 HD patients	rHuEPO + ND 50 mg IM twice weekly	rHuEPO	6 months	Hb, Ht, IGF-1	↑ Hb, ↑ IGF-1
Eiam-Ong et al. 2007 [34]	Randomized controlled trial	29 predialysis CKD	ND 100 mg/week	Conventional care	3 months	Lean body mass	↑ Lean body mass
Macdonald et al. 2007 [10]	Randomized controlled trial	54 CKD stage 5	ND 50–200 mg/week	Control	24 weeks	Appendicular lean mass	Dose-dependent ↑ lean mass
Johansen et al. 2006 [9]	2 × 2 factorial RCT	79 HD patients	ND 200 mg (men) / 100 mg (women) IM weekly ± exercise	Placebo ± exercise	12 weeks	Lean body mass, strength	↑ Lean mass; additive with exercise

Abbreviations: *ND * nandrolone decanoate, *HD*  hemodialysis, *CAPD*  continuous ambulatory peritoneal dialysis, *Hb*  hemoglobin, *Ht*  hematocrit, *RCT*  randomized controlled trial, *NR*  not reported

Of the 15 included RCTs, 12 contributed quantitative data to at least one meta-analysis (lean body mass, hemoglobin, or hematocrit). Three studies [31–33] reported relevant outcomes, primarily red cell mass, but could not be included in any quantitative synthesis. Buchwald et al. (1977) [31] was a crossover trial without suitable paired data; Solomon et al. (1988) [32] and Hendler et al. (1990) [33] did not provide extractable between-group data with measures of dispersion. These three studies are described narratively.

Eight trials were conducted before the widespread availability of recombinant erythropoietin (published between 1974 and 1990) and primarily evaluated the hematopoietic effects of nandrolone as monotherapy. The remaining seven trials were performed in the erythropoietin era; five of these assessed nandrolone either in combination with erythropoietin or resistance exercise training, or compared it directly with erythropoietin. While earlier studies focused mainly on hematologic outcomes, later trials also evaluated effects on lean body mass and functional parameters.

### Risk of bias in studies

Risk of bias was assessed using the Cochrane Risk of Bias 2 (RoB 2) tool. Overall, most studies were judged as presenting either some concerns or high risk of bias, particularly in the domains of missing outcome data and selection of the reported result. Bias in measurement of the outcome was generally rated as low risk across trials. The detailed study-level assessments and domain distributions are presented in Supplementary Figs. 1–3.

### Primary outcomes

#### Lean body mass (DXA)

**Fig. 2:**
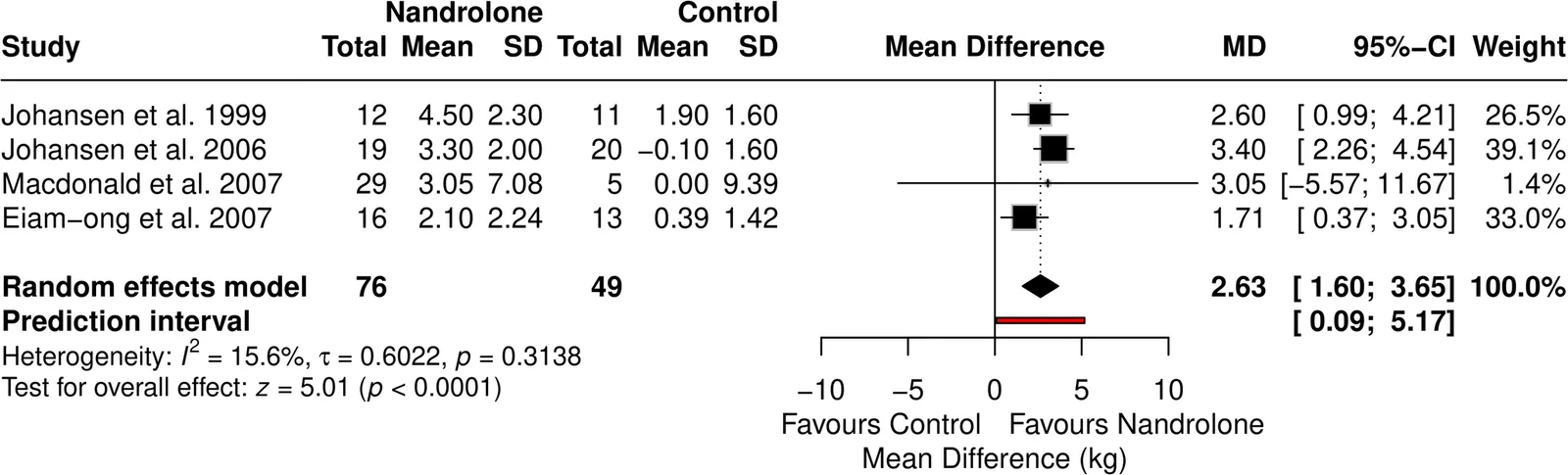
Forest plot showing the effect of nandrolone decanoate on lean body mass in adults with chronic kidney disease

Four randomized controlled trials [4, 9, 10, 34] assessed the effect of nandrolone decanoate on lean body mass (LBM) measured by dual-energy X-ray absorptiometry (DXA). A total of 76 participants received nandrolone and 49 received control interventions. Pooled analysis using a REML showed that nandrolone significantly increased LBM compared with control (mean difference [MD] 2.63 kg; 95% CI 1.60 to 3.65; *p* < 0.0001), with low between-study heterogeneity (I² = 15.6%; τ = 0.60). The 95% prediction interval ranged from 0.09 to 5.17 kg, indicating that the direction of effect consistently favors nandrolone, although the magnitude of benefit may vary across clinical settings (Fig. 2).

Sensitivity analyses demonstrated that the pooled effect on LBM remained statistically significant and of similar magnitude after excluding Johansen et al. (2006), a trial that used a 2 × 2 factorial design including resistance exercise training as a co-intervention (Supplementary Figure S4). The effect was also robust after excluding Macdonald et al. (2007), a phase II dose-finding study with a small control group (*n* = 5) (Supplementary Figure S5).

The certainty of evidence for LBM was rated as moderate, primarily due to the small number of studies and participants, along with some concerns regarding risk of bias in the included trials. Nandrolone therapy was associated with an increase in lean body mass.

#### Results of syntheses—hemoglobin

Six randomized controlled trials [35–40] contributed to the meta-analysis of hemoglobin (*n* = 239). Using a random-effects model (REML), nandrolone decanoate was associated with a pooled mean increase of 0.81 g/dL compared with control (95% CI 0.11 to 1.50; *p* = 0.0225). Between-study heterogeneity was substantial (I² = 81.3%; τ = 0.73; *p* < 0.0001), and the 95% prediction interval was wide (−1.27 to 2.89 g/dL), indicating that the expected effect in a future comparable setting could range from a decrease to a clinically relevant increase. Although the pooled estimate favored nandrolone, both the magnitude and the direction of effect remain uncertain across clinical contexts.

**Fig. 3:**
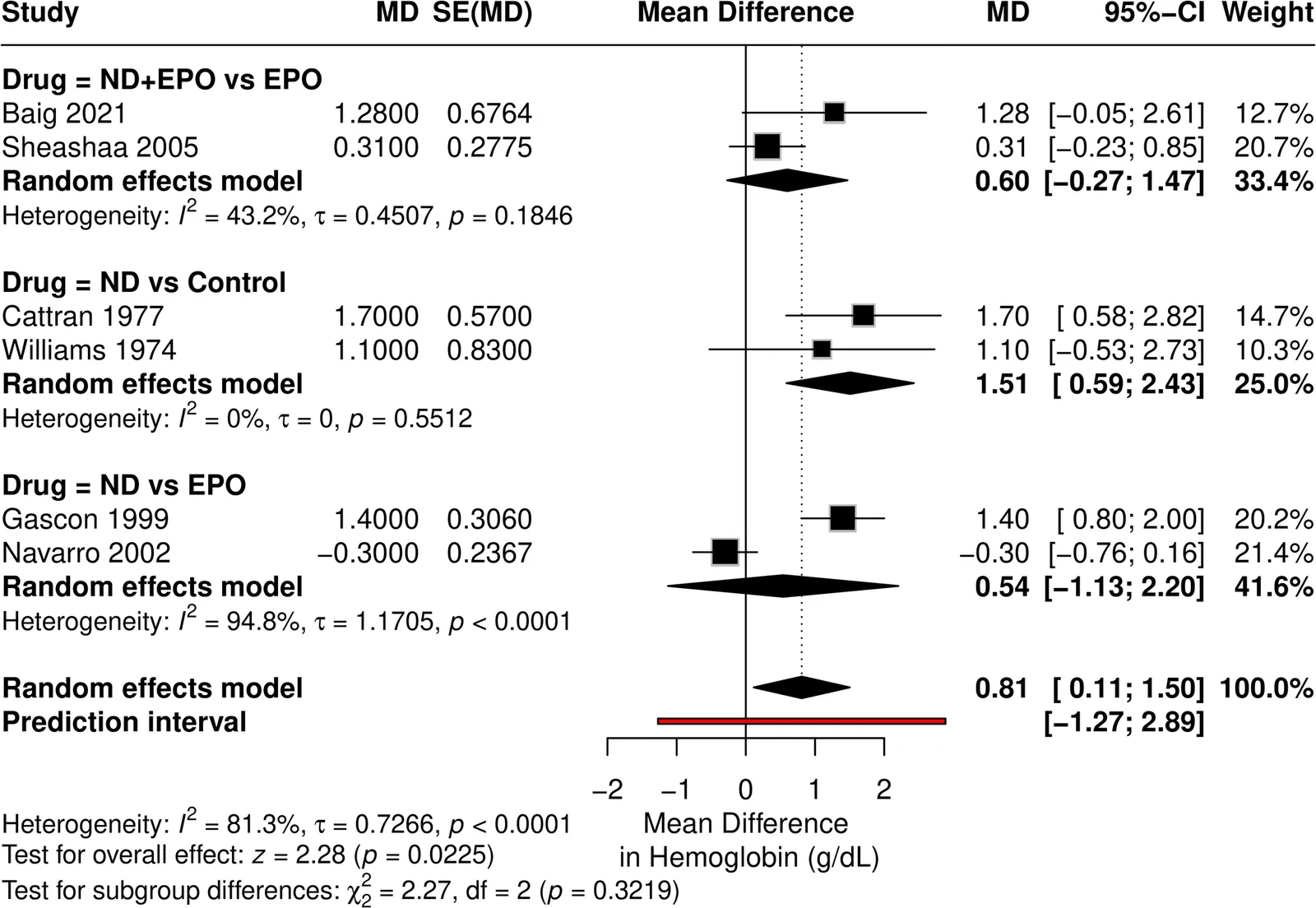
Random-effects meta-analysis of the effect of nandrolone decanoate on hemoglobin (g/dL), stratified by comparator type, in adults with chronic kidney disease

Subgroup analyses according to comparator type showed variable estimates. When nandrolone was combined with erythropoietin and compared with erythropoietin alone, the mean difference was 0.60 g/dL (95% CI − 0.27 to 1.47; I² = 43.2%). Trials comparing nandrolone with control or placebo showed a larger increase (MD 1.51 g/dL; 95% CI 0.59 to 2.43; I² = 0%). Studies comparing nandrolone directly with erythropoietin yielded a smaller, non-significant effect (MD 0.54 g/dL; 95% CI − 1.13 to 2.20; I² = 94.8%). The test for subgroup differences was not statistically significant (χ² = 2.27, df = 2; *p* = 0.3219), suggesting that comparator type did not fully explain the observed heterogeneity (Fig. 3).

Two additional studies were not included in the quantitative synthesis. Buchwald et al. [31], a double-blind crossover trial, reported increases in hemoglobin during nandrolone treatment but could not be pooled because of the crossover design and lack of suitable paired data. Solomon et al. (1988)³⁴ described improvements in hemoglobin but did not provide extractable between-group data with measures of dispersion.

Sensitivity analyses were performed to assess robustness. Exclusion of Navarro et al. [39] increased the pooled effect (MD 1.07 g/dL; 95% CI 0.47 to 1.68) and reduced heterogeneity (I² = 57.8%) (Supplementary Figure S6). Exclusion of the crossover trial [36] resulted in loss of statistical significance (MD 0.65 g/dL; 95% CI − 0.08 to 1.38; *p* = 0.0809), with heterogeneity remaining high (Supplementary Figure S7). These findings indicate that the hematologic effect of nandrolone on hemoglobin is sensitive to study design and population characteristics.

The certainty of evidence for hemoglobin was rated as very low, primarily because of substantial heterogeneity, imprecision (wide prediction interval), and serious concerns regarding risk of bias. Most trials presented some concerns or high risk of bias, particularly in the domains of missing outcome data and selection of the reported result. These limitations substantially reduce confidence in the pooled estimate.

#### Results of syntheses—hematocrit

Seven randomized controlled trials [8, 35–37, 39–41] contributed to the meta-analysis of hematocrit. Using a random-effects model (REML), nandrolone decanoate was associated with a pooled mean increase of 3.12% compared with control (95% CI 1.30 to 4.94; *p* = 0.0008). However, substantial between-study heterogeneity was observed (I² = 87.2%; τ = 2.22), and the 95% prediction interval was wide (−2.77% to 9.00%), indicating that both the magnitude and potentially the direction of the effect may vary considerably across clinical contexts.

**Fig. 4:**
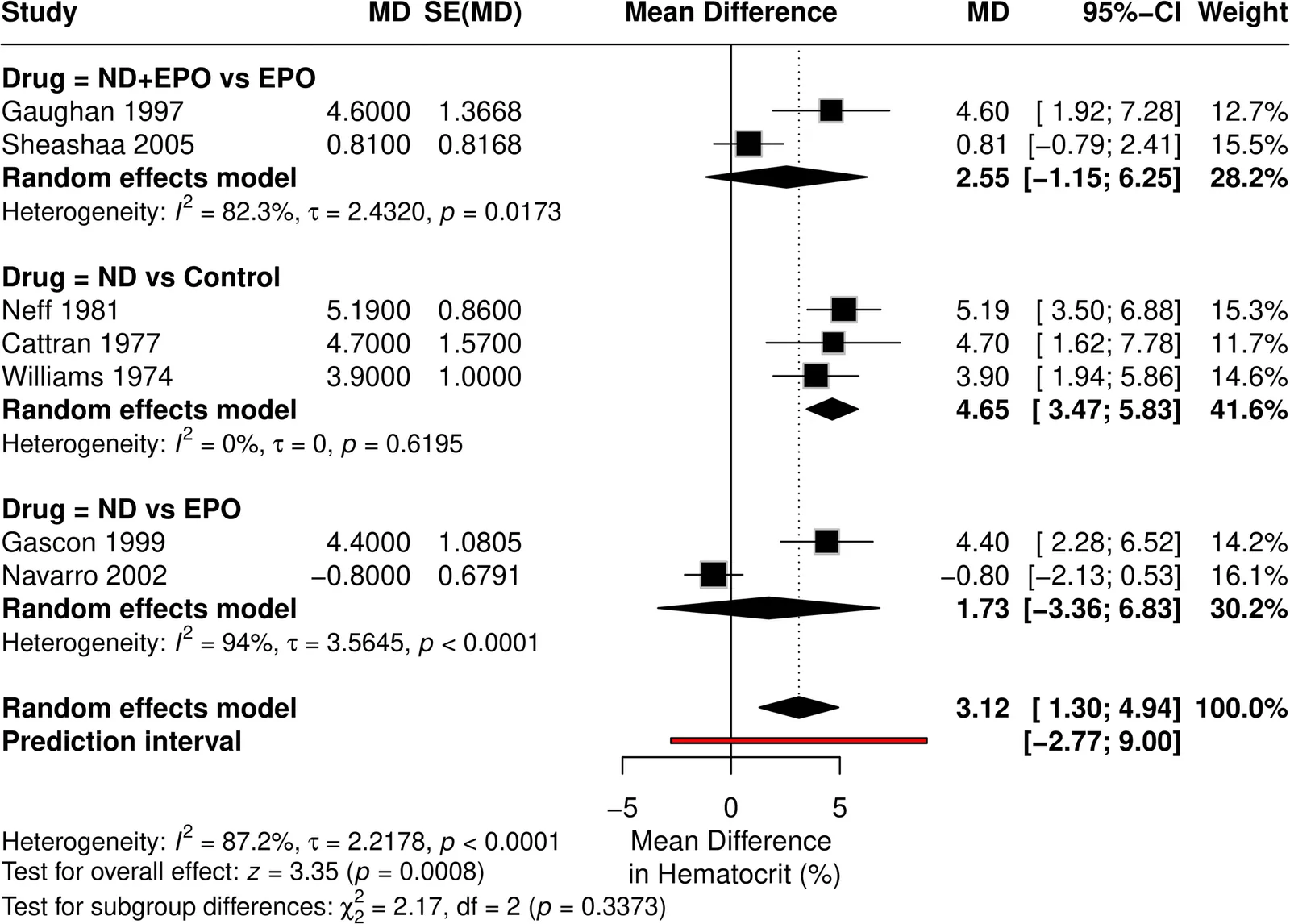
Random-effects meta-analysis of the effect of nandrolone decanoate on hematocrit (%), stratified by comparator type, in adults with chronic kidney disease

Subgroup analyses according to comparator type showed variable effect estimates. When nandrolone was combined with erythropoietin and compared with erythropoietin alone, the pooled mean difference was 2.55% (95% CI − 1.15 to 6.25), with substantial heterogeneity (I² = 82.3%). In trials comparing nandrolone with control or placebo, a statistically significant increase was observed (MD 4.65%; 95% CI 3.47 to 5.83), with no heterogeneity (I² = 0%). Studies comparing nandrolone directly with erythropoietin also favored nandrolone (MD 4.40%; 95% CI 2.28 to 6.52), although heterogeneity was substantial (I² = 94%). The test for subgroup differences was not statistically significant (χ² = 2.17, df = 2; *p* = 0.3373), suggesting that comparator type did not fully explain the observed heterogeneity (Fig. 4).

Two additional studies were not included in the quantitative synthesis. Buchwald et al. (1977), a double-blind crossover trial, reported improvements in packed cell volume and red cell mass with nandrolone but could not be pooled due to its crossover design. Hendler et al. (1990) [33] also described increases in red cell mass but did not provide sufficient extractable data for meta-analysis.

Sensitivity analyses were performed to assess the robustness of the findings. Exclusion of Navarro et al. [39], a trial conducted in elderly male patients on peritoneal dialysis, resulted in a larger pooled effect (MD 3.81%; 95% CI 2.35 to 5.28) with reduced but still substantial heterogeneity (I² = 70.1%) (Supplementary Figure S8). Exclusion of crossover trials also yielded a statistically significant increase in hematocrit (MD 2.44%; 95% CI 0.25 to 4.63), although heterogeneity remained substantial (Supplementary Figure S9). These results suggest that the effect of nandrolone on hematocrit is relatively consistent across different study designs and populations.

The certainty of evidence for hematocrit was rated as very low, mainly because of substantial heterogeneity, imprecision, and risk of bias. The majority of included studies were judged as having some concerns or high risk of bias, especially regarding missing outcome data and selective reporting. These methodological limitations warrant a cautious interpretation of the findings.

### Secondary outcomes

#### Quality of life

Two randomized controlled trials [4, 9] evaluated the effects of nandrolone decanoate on quality of life in patients with chronic kidney disease. In a double-blind placebo-controlled trial, Johansen et al. (1999) [4] found that nandrolone decanoate significantly reduced fatigue scores after 24 weeks (*P* = 0.04), but did not improve the SF-36 Physical Function domain. In a subsequent 2 × 2 factorial trial, Johansen et al. (2006) [9] observed that resistance training improved the SF-36 Physical Function domain (*P* = 0.03), whereas nandrolone alone did not produce significant changes in any quality-of-life subscale compared with placebo.

Overall, the available evidence suggests that nandrolone decanoate has limited and inconsistent effects on patient-reported outcomes. While one study showed a reduction in fatigue, improvements in functional domains of quality of life were not observed. Both trials were limited by small sample sizes, short follow-up periods, and the absence of CKD-specific quality-of-life instruments or comprehensive assessments of physical function and frailty. Therefore, it remains uncertain whether the increases in lean body mass translate into meaningful benefits in quality of life or daily functioning (Table 3).

**Table 3:** Effects of nandrolone decanoate on quality of life in chronic kidney disease

Study	Sample Size (*N*)	Population	Intervention	Dose	Duration	Quality of Life Assessment	Main Results
Johansen et al. 1999 [4]	29	Hemodialysis (HD) and CAPD patients with malnutrition	Nandrolone decanoate vs. placebo	100 mg/week IM	24 weeks	SF-36 (Physical Function), fatigue assessment	Significant reduction in fatigue in the nandrolone group (*P* = 0.04); no significant change in SF-36 Physical Function.
Johansen et al. 2006 [9]	79	Hemodialysis patients with sarcopenia and muscle weakness	Nandrolone alone, resistance training (RT) alone, nandrolone + RT, placebo	100–200 mg/week IM	12 weeks	SF-36 Physical Function	Resistance training significantly improved Physical Function (*P* = 0.03); nandrolone alone did not produce significant change.

#### Safety

Adverse events were reported in most of the included trials. No severe or life-threatening adverse events directly attributed to nandrolone decanoate were consistently observed. The most commonly reported adverse events were androgenic effects, including acne, hirsutism, amenorrhea, and virilization, particularly among female participants. Other frequently reported events included local injection-site reactions and mild elevations in liver enzymes. Lipid profile alterations, mainly increased triglycerides and decreased HDL cholesterol, were also documented in several studies.

Withdrawals due to adverse events occurred predominantly in women, most often due to virilization or menstrual disturbances. One case of vascular access thrombosis was reported in a single trial, although causality could not be confirmed. Detailed information on adverse events is presented in Table 4.

**Table 4:** Adverse events reported in randomized trials of nandrolone decanoate in chronic kidney disease

Study	*N*	Population	Reported Adverse Events	Withdrawals Due to AE	Severe Events
Cattran et al. 1977 [36]	37	Hemodialysis	↑ Triglycerides (+ 53 mg/dL); local hematomas	None	None
Johansen et al. 1999 [4]	29	HD/CAPD	Local hematomas; amenorrhea; acne; testicular size reduction; no significant LFT changes	2 women (amenorrhea/acne)	None
Johansen et al. 2006 [9]	79	Hemodialysis	Mild androgenic symptoms; sexual dysfunction; slight aggression increase	2 (sexual side effects/fear of AE)	None
Macdonald et al. 2007 [10]	54	HD/PD	ALT elevation (5/18 high-dose); water retention; carpal tunnel; mild hypertension; PSA rise	4 (virilization)	1 vascular access thrombosis (not confirmed related)
Gascón et al. 1999 [37]	33	Elderly HD	↑ Triglycerides; ↓ HDL cholesterol	None	None
Sheashaa et al. 2005 [40]	32	HD, EPO-resistant	Hirsutism; acne; ↑ LFTs	4 women (hirsutism/LFT elevation)	None
Gaughan et al. 1997 [41]	19	HD, EPO-treated	Local injection site pain	None	None
Hendler & Solomon, 1990 [33]	9	Hemodialysis	Mild acne	None	None

#### Publication bias

Assessment of publication bias through funnel plot inspection and Egger’s regression test was not performed for any outcome, as none of the meta-analyses included 10 or more studies. Specifically, hemoglobin (*n* = 6) and hematocrit (*n* = 7) did not meet the recommended threshold for reliable statistical evaluation of small-study effects.

Given the limited number of trials and the predominance of small, single-center studies, the possibility of publication bias or selective reporting cannot be excluded.

#### Certainty of the evidence (GRADE)

The certainty of evidence was assessed using the GRADE approach. Summary of Findings tables are presented in Supplementary Table S3.

## Discussion

This systematic review and meta-analysis synthesized randomized controlled trials evaluating the anabolic and hematologic effects of nandrolone decanoate in adults with chronic kidney disease. The pooled results demonstrate that nandrolone is associated with significant increases in lean body mass and with higher mean hemoglobin and hematocrit levels compared with control conditions. However, substantial heterogeneity, very low certainty of evidence for hematologic outcomes, and wide prediction intervals limit confidence in a consistent or generalizable hematologic benefit across clinical contexts.

Several clinical and methodological factors likely contributed to the heterogeneity observed in hematologic outcomes. The included trials enrolled diverse CKD populations across different disease stages and dialysis modalities, with variable inflammatory burden, baseline anemia severity, and concomitant therapies such as erythropoiesis-stimulating agents and iron supplementation [42, 43]. Differences in intervention duration, dosing regimens, and nutritional status, including protein-energy wasting, may also have influenced responsiveness [14, 15]. These sources of variability likely explain the wide prediction intervals and underscore the context-dependent nature of hematologic effects.

In contrast, the anabolic effect on lean body mass warrants particular attention. This finding is clinically relevant in the context of the high burden of protein-energy wasting and sarcopenia in CKD, both strongly associated with increased morbidity and mortality [2, 14, 15]. In chronic disease settings, increases in lean mass have been linked to improvements in muscle strength and physical performance [44, 45]. These findings are consistent with a recent umbrella review of β-hydroxy-β-methyl butyrate (HMB) supplementation, which demonstrated significant improvements in muscle mass and fat-free mass across various populations, supporting the anabolic potential of interventions targeting muscle protein metabolism in chronic conditions [46]. Although functional outcomes were inconsistently reported, the anabolic effect observed raises the possibility that gains in muscle mass could translate into improvements in physical function and quality of life in selected CKD populations [47].

The anabolic response is biologically plausible in CKD, a condition characterized by chronic inflammation, endocrine dysfunction, metabolic derangements, and anabolic resistance [48]. Functional hypogonadism and low testosterone levels are common and have been associated with lower hemoglobin levels and increased cardiovascular and all-cause mortality [7]. Nandrolone decanoate, an anabolic steroid with moderate androgenic activity, has historically been explored as an adjunctive strategy to counteract catabolism and potentially support erythropoiesis [11]. Earlier trials emphasized hematopoietic outcomes before the widespread availability of recombinant human erythropoietin [8, 35, 36], whereas later studies focused more on body composition in patients with wasting phenotypes [4, 9, 10, 34].

An important consideration is that the evidence base is largely historical. Most trials were published before 2008, with only one additional trial published in 2021. Therefore, most available evidence predates current standards of CKD anemia management, nutritional care, and body composition assessment. Over the past two decades, anemia treatment has evolved with optimized erythropoiesis-stimulating agent protocols, improved iron management, and the introduction of hypoxia-inducible factor prolyl hydroxylase inhibitors [49, 50]. Advances in nutritional assessment, structured protein supplementation, and standardized diagnostic frameworks for protein-energy wasting and sarcopenia have also reshaped management strategies [51, 52]. Consequently, the baseline clinical profile and therapeutic responsiveness of patients with CKD today differ substantially from those enrolled in earlier nandrolone trials.

In the current therapeutic landscape, the incremental value of nandrolone relative to optimized ESA regimens, HIF-PHIs, and comprehensive nutritional management remains uncertain [13, 43, 53]. The absence of contemporary trials conducted under modern standards of care further limits confidence in its incremental benefit [52, 53]. Rather than positioning nandrolone as an alternative to contemporary standards of care, any potential role would be restricted to highly selected subgroups (e.g., ESA-resistant anemia associated with inflammation-mediated hyporesponsiveness [13, 43] or severe protein-energy wasting refractory to optimized nutritional support [14, 16, 51]. These hypotheses require confirmation in adequately powered contemporary randomized trials conducted under current standards of care.

For hematologic outcomes, pooled estimates favored nandrolone, but substantial heterogeneity and wide prediction intervals indicate that the magnitude of effect may vary across clinical settings. Although partial resistance to erythropoiesis-stimulating agents is common in CKD [8, 41], the available evidence supporting nandrolone in this context remains heterogeneous and largely derived from small, methodologically limited trials [11, 16].

Patient-reported outcomes were sparsely assessed. Evidence for quality-of-life improvement was limited to a small number of trials, with signals of reduced fatigue in some contexts but less consistent effects on broader physical function domains [4, 9]. Overall, the current literature does not allow firm conclusions regarding the impact of nandrolone on quality of life, as available trials were small and primarily focused on hematologic outcomes [11]. This highlights the need for future studies to incorporate validated patient-centered endpoints, including quality of life and functional measures, alongside body composition and hematologic parameters [54], in line with contemporary recommendations for CKD research [55, 56].

Across included trials, adverse events were generally described as mild to moderate. The most frequently reported adverse effects were androgenic, including acne, hirsutism, amenorrhea, and virilization, particularly among women [4, 10, 11, 36, 37, 40]. Other commonly observed events included local injection-site reactions and mild, usually reversible, elevations in liver enzymes. Lipid profile changes, mainly increased triglycerides and decreased HDL cholesterol, were also documented in several studies. However, these findings must be interpreted with considerable caution. Most trials were small and had short follow-up periods. Adverse event collection and reporting standards were substantially less rigorous than current practice, and none of the included studies were adequately powered to detect rare but clinically important adverse events. Furthermore, long-term data on cardiovascular, metabolic, hepatic, and endocrine safety are lacking. Therefore, while short-term tolerability appeared acceptable in the context of these historical trials, the long-term safety profile of nandrolone decanoate in patients with chronic kidney disease remains insufficiently characterized and cannot be reliably established based on the available evidence.

The evidence base remains largely historical. The meta-analysis of lean body mass included only four trials (*n* = 125). Few studies reported validated patient-centered outcomes (muscle strength, physical function, hospitalization, or mortality), so it remains uncertain whether increases in lean body mass translate into meaningful clinical benefits. Substantial clinical and statistical heterogeneity, particularly for hematologic outcomes, and the very low certainty of evidence for hemoglobin and hematocrit further warrant cautious interpretation.

From a methodological perspective, substantial clinical and statistical heterogeneity was present across trials, particularly for hematologic outcomes, and the small number of studies in each subgroup limited our ability to explore potential effect modifiers through meta-regression or adequately powered subgroup analyses. Although formal assessment of publication bias was not feasible, the predominance of small, single-center trials increases the possibility of small-study effects. Furthermore, long-term safety data were limited, and most studies were not powered to detect rare but clinically important adverse events. These factors, combined with the very low certainty of evidence for hemoglobin and hematocrit, warrant cautious interpretation of the pooled estimates.

This review has several important strengths. It represents the first systematic review and meta-analysis specifically focused on nandrolone decanoate in chronic kidney disease, rather than pooling heterogeneous androgenic agents. By restricting inclusion to randomized controlled trials, the present synthesis provides a higher level of internal validity and minimizes confounding inherent to observational designs. In addition, the quantitative pooling of lean body mass, hemoglobin, and hematocrit offers a more precise estimate of the anabolic and hematologic effects of nandrolone across different CKD settings. The use of random-effects models, assessment of heterogeneity, and incorporation of prediction intervals further strengthen the robustness and clinical interpretability of the findings. Finally, safety outcomes were systematically summarized, allowing a balanced evaluation of potential benefits and risks. By integrating quantitative synthesis with contextual clinical interpretation, this review provides a comprehensive framework for evaluating the potential role of nandrolone in CKD.

## Conclusion

Nandrolone decanoate was associated with increases in lean body mass in adults with chronic kidney disease. In contrast, pooled estimates for hemoglobin and hematocrit were limited by substantial heterogeneity and very low certainty of evidence, precluding firm conclusions regarding hematologic benefit.

In current CKD practice, nandrolone should not be considered first-line therapy for anemia. Its potential role, if any, remains uncertain and would be limited to selected patients with refractory anemia or severe protein-energy wasting despite optimized conventional care. Short-term safety appeared acceptable in historical trials, but long-term cardiovascular, metabolic, and endocrine risks are poorly characterized. Whether increases in lean body mass translate into patient-centered benefits is also uncertain.

Well-designed contemporary head-to-head randomized trials are needed to determine whether nandrolone has any role in modern CKD management.

## Supplementary information

Below is the link to the electronic supplementary material.

Supplementary Material 1
