# Supplementary material for: Effects of nandrolone decanoate on lean body mass and anemia in adults with chronic kidney disease: a systematic review and meta-analysis
Source: Endocrine. 2026 Sep 9;91(1):294. doi: 10.1007/s12020-026-04771-9 (PMC13558390; doi:10.1007/s12020-026-04771-9)
Supplement: Supplementary file 1 [file 12020_2026_4771_MOESM1_ESM.docx]

**Supplementary Material**

**Full electronic search strategies**

**Supplementary Table 1.** Comprehensive electronic search strategy

| **Database** | **Search Strategy** |
| --- | --- |
| PubMed/MEDLINE | ("Nandrolone"[Mesh] OR "19-Nortestosterone" OR "17beta-Hydroxy-19-Nor-4-Androsten-3-One" OR "17beta Hydroxy 19 Nor 4 Androsten 3 One" OR Estrenolone OR Nortestosterone OR "17-Hydroxy-Estr-4-Ene-3-One" OR Norandrostenolone OR "17 beta Hydroxyestr 4 en 3 one decanoate" OR "19 Nortestosterone Decanoate" OR "19 nor 4 Androstene 17 beta ol 3 one 17 decanoate" OR Retabolil OR Retabolyl OR Decadurabolin OR Decadurobolin) AND ("Renal Insufficiency, Chronic"[Mesh] OR "Kidney Failure, Chronic"[Mesh] OR "Renal Dialysis"[Mesh]) AND (randomized controlled trial[pt] OR controlled clinical trial[pt] OR randomized[tiab] OR placebo[tiab] OR drug therapy[sh] OR randomly[tiab] OR trial[tiab] OR groups[tiab] NOT (animals[mh] NOT humans[mh])) |
| Embase | ('nandrolone'/exp OR '19 nortestosterone' OR '17beta hydroxy 19 nor 4 androsten 3 one' OR 'estrenolone' OR 'nortestosterone' OR 'norandrostenolone' OR 'nandrolone decanoate' OR '19 nortestosterone decanoate' OR retabolil OR retabolyl OR decadurabolin OR decadurobolin) AND ('chronic kidney failure'/exp OR 'chronic kidney disease':ti,ab OR ckd:ti,ab OR 'chronic renal failure':ti,ab) AND ('randomized controlled trial'/exp OR 'controlled clinical trial'/exp OR random*:ti,ab OR placebo*:ti,ab OR 'drug therapy'/exp OR trial*:ti,ab OR group*:ti,ab) AND [humans]/lim; #1 AND [embase]/lim NOT ([embase]/lim AND [medline]/lim) |
| Cochrane CENTRAL | (nandrolone OR "19 nortestosterone" OR "17beta hydroxy 19 nor 4 androsten 3 one" OR estrenolone OR nortestosterone OR norandrostenolone OR "nandrolone decanoate" OR "19 nortestosterone decanoate" OR retabolil OR retabolyl OR decadurabolin OR decadurobolin) AND ("kidney failure chronic" OR "chronic kidney disease" OR CKD OR "chronic renal failure") |
| LILACS | (nandrolona OR nandrolone OR nortestosterona OR nortestosterone OR "nandrolona decanoato" OR "nandrolone decanoate" OR retabolil OR retabolyl OR decadurabolin OR decadurobolin) AND ("insuficiencia renal cronica" OR "doenca renal cronica" OR "falencia renal cronica" OR "chronic kidney disease" OR "chronic renal failure" OR CKD) |

Searches performed on February 18, 2026.

**Full text excluded studies with reasons**

**Supplementary Table 2.** Full-text exclusions and reasons for exclusion

| **Study** | **Year** | **Study design** | **Reason for exclusion** |
| --- | --- | --- | --- |
| Doane et al. | 1975 | Prospective uncontrolled study | Not a randomized controlled trial |
| Teruel et al. | 1997 | Prospective single-arm study | Not randomized; no control group |
| Ballal et al. | 1991 | Non-randomized comparative study | Did not meet RCT inclusion criteria |
| Lee et al. | 2002 | Non-randomized interventional study | Did not meet RCT inclusion criteria |
| Johansen | 2001 | Editorial/Review | Not original RCT data |

**Outcome-specific risk of bias assessment (RoB 2)**

**Supplementary Figure S1.** Risk of bias summary for lean body mass (RoB 2) in randomized trials of nandrolone decanoate.

**Supplementary Figure S2.** Risk of bias summary for hemoglobin (RoB 2) in randomized trials of nandrolone decanoate.

**Supplementary Figure S3.** Risk of bias summary for hematocrit (RoB 2) in randomized trials of nandrolone decanoate.

**Supplementary Figure S4.** Sensitivity analysis excluding Johansen et al. (2006) for the effect of nandrolone decanoate on lean body mass (kg) in adults with chronic kidney disease.

**Supplementary Figure S5.** Sensitivity analysis excluding Macdonald et al. (2007) for the effect of nandrolone decanoate on lean body mass (kg) in adults with chronic kidney disease.

**Supplementary Figure S6.** Sensitivity analysis excluding Navarro et al. (2002) for the effect of nandrolone decanoate on hemoglobin (g/dL) in adults with chronic kidney disease.

**Supplementary Figure S7.** Sensitivity analysis excluding Cattran et al. (1977) for the effect of nandrolone decanoate on hemoglobin (g/dL) in adults with chronic kidney disease.

**Supplementary Figure S8.** Sensitivity analysis excluding Navarro et al. (2002) for the effect of nandrolone decanoate on hematocrit (%) in adults with chronic kidney disease

**Supplementary Figure S9.** Sensitivity analysis excluding crossover trials for the effect of nandrolone decanoate on hematocrit (%) in adults with chronic kidney disease.

**Certainty of the Evidence (GRADE)**

**Supplementary Table 3.** Summary of Findings (GRADE): Nandrolone Decanoate for Adults with Chronic Kidney Disease

| **Nandrolone decanoate compared to placebo or standard care for adults with chronic kidney disease** | | | | | | |
| --- | --- | --- | --- | --- | --- | --- |
| **Patient or population:** **Adults with chronic kidney disease (including hemodialysis, peritoneal dialysis, or advanced predialysis)**  **Setting: Outpatient nephrology and dialysis care settings**  **Intervention:** Intramuscular nandrolone decanoate (any dose or duration)  **Comparison:** Placebo or standard care without nandrolone | | | | | | |
| Outcome № of participants (studies) | Relative effect (95% CI) | **Anticipated absolute effects (95% CI)** | | | Certainty | What happens |
|  |  | **[comparison]** | **[intervention]** | **Difference** |  |  |
| Lean Body Mass assessed with: Kg № of participants: 125 (4 RCTs) | - |  | - | MD **2.63 kg higher** (1.6 higher to 3.65 higher) | ⨁⨁⨁◯ Moderate^a^ | Nandrolone decanoate probably increases lean body mass in adults with chronic kidney disease. |
| Hemoglobin assessed with: g/dL № of participants: 239 (6 RCTs) | - |  | - | MD **0.81 g/dL higher** (0.11 higher to 1.5 higher) | ⨁◯◯◯ Very low^b,c,d^ | The effect of nandrolone on hemoglobin levels is very uncertain in adults with chronic kidney disease. |
| Hematocrit assessed with: % № of participants: 198 (7 RCTs) | - |  | - | MD **3.12 % higher** (1.3 higher to 4.94 higher) | ⨁◯◯◯ Very low^e,f,g^ | The effect of nandrolone on hematocrit levels is very uncertain in adults with chronic kidney disease. |
| ***The risk in the intervention group** (and its 95% confidence interval) is based on the assumed risk in the comparison group and the **relative effect** of the intervention (and its 95% CI).  **CI:** confidence interval; **MD:** mean difference | | | | | | |
| **GRADE Working Group grades of evidence** **High certainty:** we are very confident that the true effect lies close to that of the estimate of the effect. **Moderate certainty:** we are moderately confident in the effect estimate: the true effect is likely to be close to the estimate of the effect, but there is a possibility that it is substantially different. **Low certainty:** our confidence in the effect estimate is limited: the true effect may be substantially different from the estimate of the effect. **Very low certainty:** we have very little confidence in the effect estimate: the true effect is likely to be substantially different from the estimate of effect. | | | | | | |

**Explanations**

a. The certainty of evidence for lean body mass was downgraded by one level for risk of bias because several trials presented “some concerns” in key RoB 2 domains (particularly randomization process and potential deviations from intended interventions and/or incomplete outcome data). Given that lean body mass was objectively measured by DXA and results were consistent, no further downgrades were applied.

b. The evidence was downgraded by one level for risk of bias because several included trials presented methodological limitations, including unclear allocation concealment, potential deviations from intended interventions, and incomplete outcome reporting.

c. The evidence was downgraded for inconsistency due to substantial between-study heterogeneity (I² = 81.3%) and wide prediction intervals crossing the line of no effect, suggesting variability in treatment response across clinical contexts.

d. The evidence was downgraded for imprecision because, despite statistical significance, the overall sample size was relatively small and the prediction interval included the possibility of no effect, indicating uncertainty regarding the magnitude of benefit in future populations.

e. The certainty of evidence was downgraded by one level for risk of bias because several included trials presented methodological limitations, including unclear random sequence generation and allocation concealment, potential deviations from intended interventions (particularly related to concomitant erythropoietin use), incomplete outcome data reporting, and absence of prospectively registered protocols in older studies.

f. The certainty of evidence was downgraded for inconsistency due to substantial between-study heterogeneity (I² = 87.2%) and wide prediction intervals crossing the line of no effect, indicating considerable variability in treatment effects across clinical settings and comparator groups.

g. The certainty of evidence was downgraded for imprecision because, despite statistical significance, the overall sample size was limited and the prediction interval included the possibility of no effect, indicating uncertainty about the magnitude and direction of the effect in future populations.
